# Supplementary material for: A Custom Microcontrolled and Wireless-Operated Chamber for Auditory Fear Conditioning
Source: Front Neurosci. 2019 Nov 7;13:1193. doi: 10.3389/fnins.2019.01193 (PMC6853868; doi:10.3389/fnins.2019.01193)
Supplement: Supplementary file 1 [file Data_Sheet_1.pdf]

A Custom Microcontrolled and Wireless-Operated Chamber for Auditory Fear Conditioning

Authors: Paulo Aparecido Amaral Júnior, Flávio Afonso Gonçalves Mourão, Mariana Chamon Ladeira Amâncio, Hyorrana Pereira Pinto, Vinícius Rezende Carvalho, Leonardo de Oliveira Guarnieri, Hermes Aguiar Magalhães, Márcio Flávio Dutra Moraes

INSTRUCTIONS

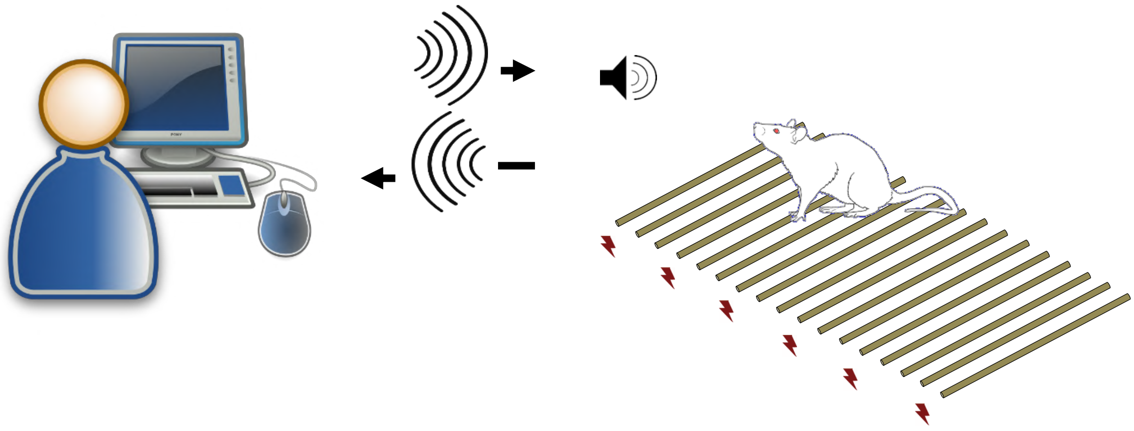

NNC

Assembly Instructions

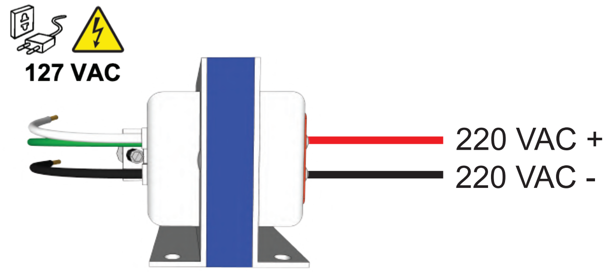

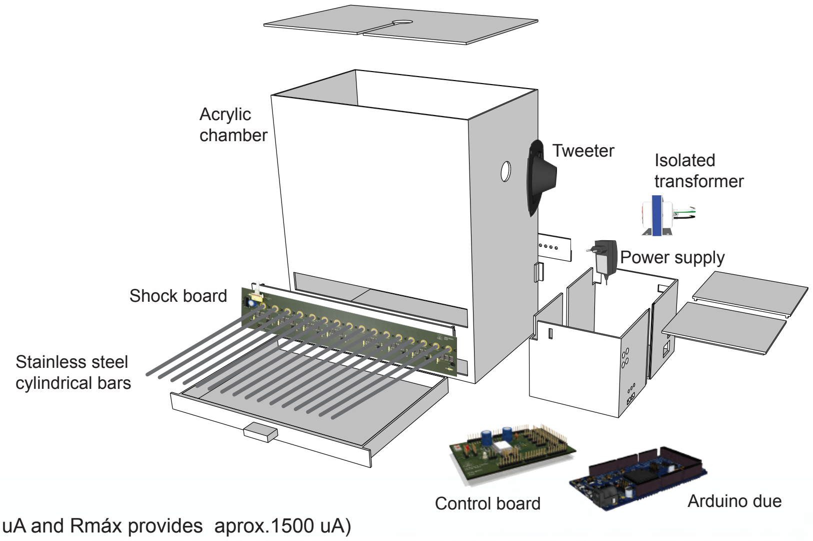

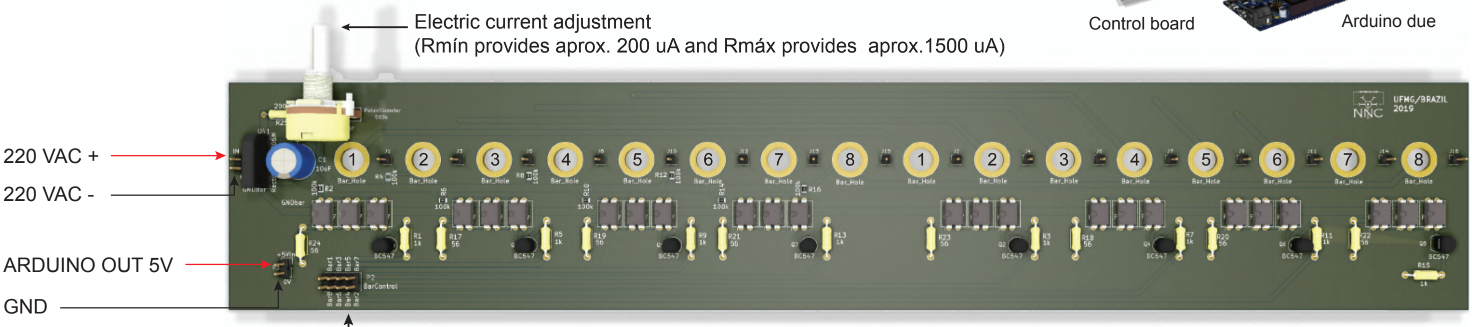

Buid in KiCad EDA 5.1.2 (http://www.kicad-pcb.org/)

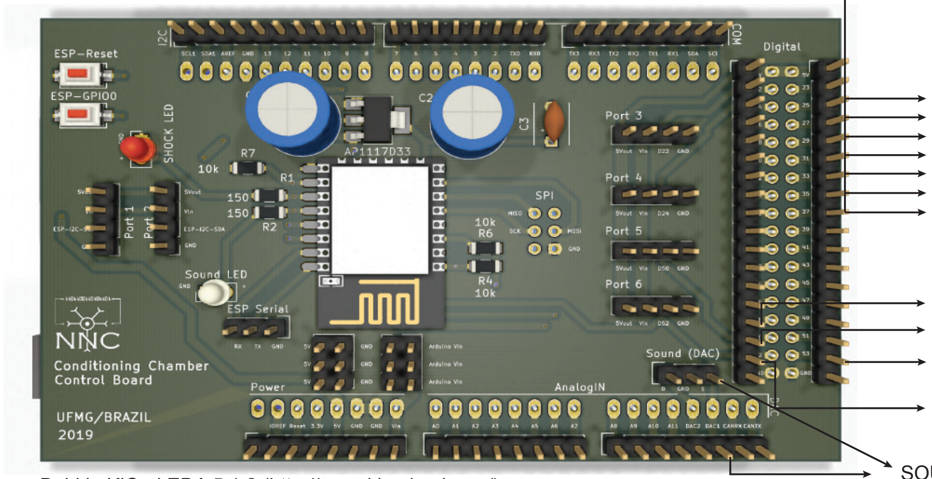

OUTPUT BARS / PULSE CONTROL

BAR 1 (PIN 23)
BAR 2 (PIN 25)
BAR 3 (PIN 27)
BAR 4 (PIN 29)
BAR 5 (PIN 31)
BAR 6 (PIN 33)
BAR 7 (PIN 35)
BAR 8 (PIN 37)

DIGITAL PINS FOR EXTERNAL CONTROL

Input pin (48) used to Abort experiment (Abort).
Input pin (50) by which ESP8266 controls SOUND
Output pin (53) used to generate a reference signal (a square wave) that represents sound modulator.
Input pin (52) by which ESP8266 controls SHOCK.

SOUND OUTPUT

Buid in KiCad EDA 5.1.2 (http://www.kicad-pcb.org/)

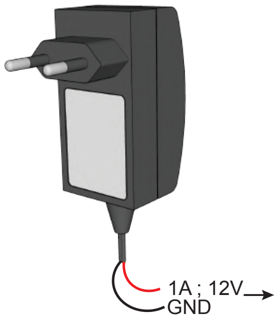

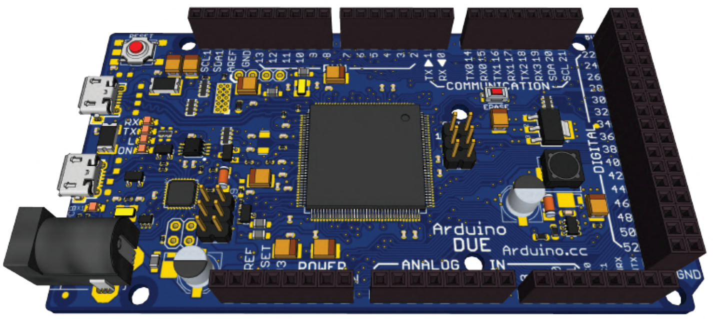

https://store.arduino.cc/usa/due
https://3dwarehouse.sketchup.com/model/uef043628-8edb-4632-96bb-0f367bee29d8/Arduino-DUE?hl=en

Suggestion:
PAM8610 10W
STEREO AUDIO AMPLIFIER MODULE

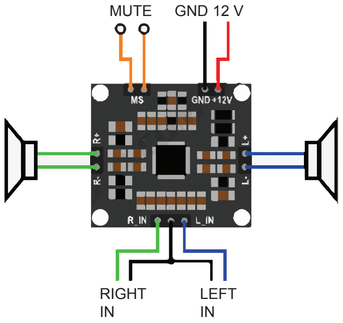

in this case a power source > 1A is required

https://hobbycomponents.com/audio/664-pam8610-10w-stereo-audio-amplifier-module

Original bar configuration for experiments with rats

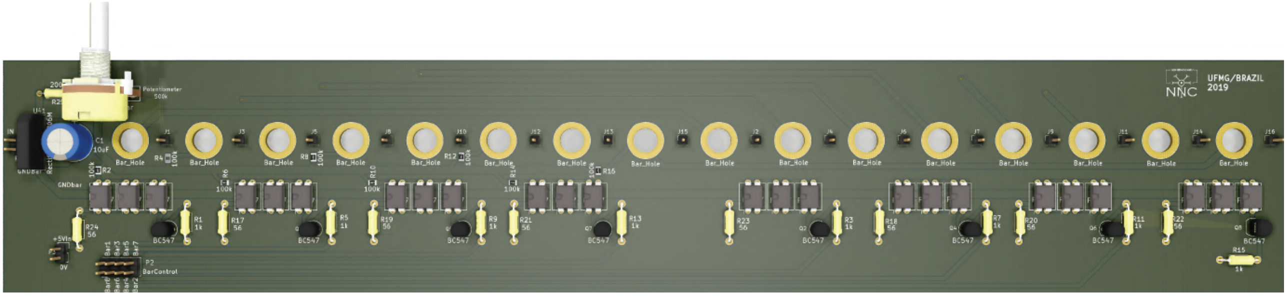

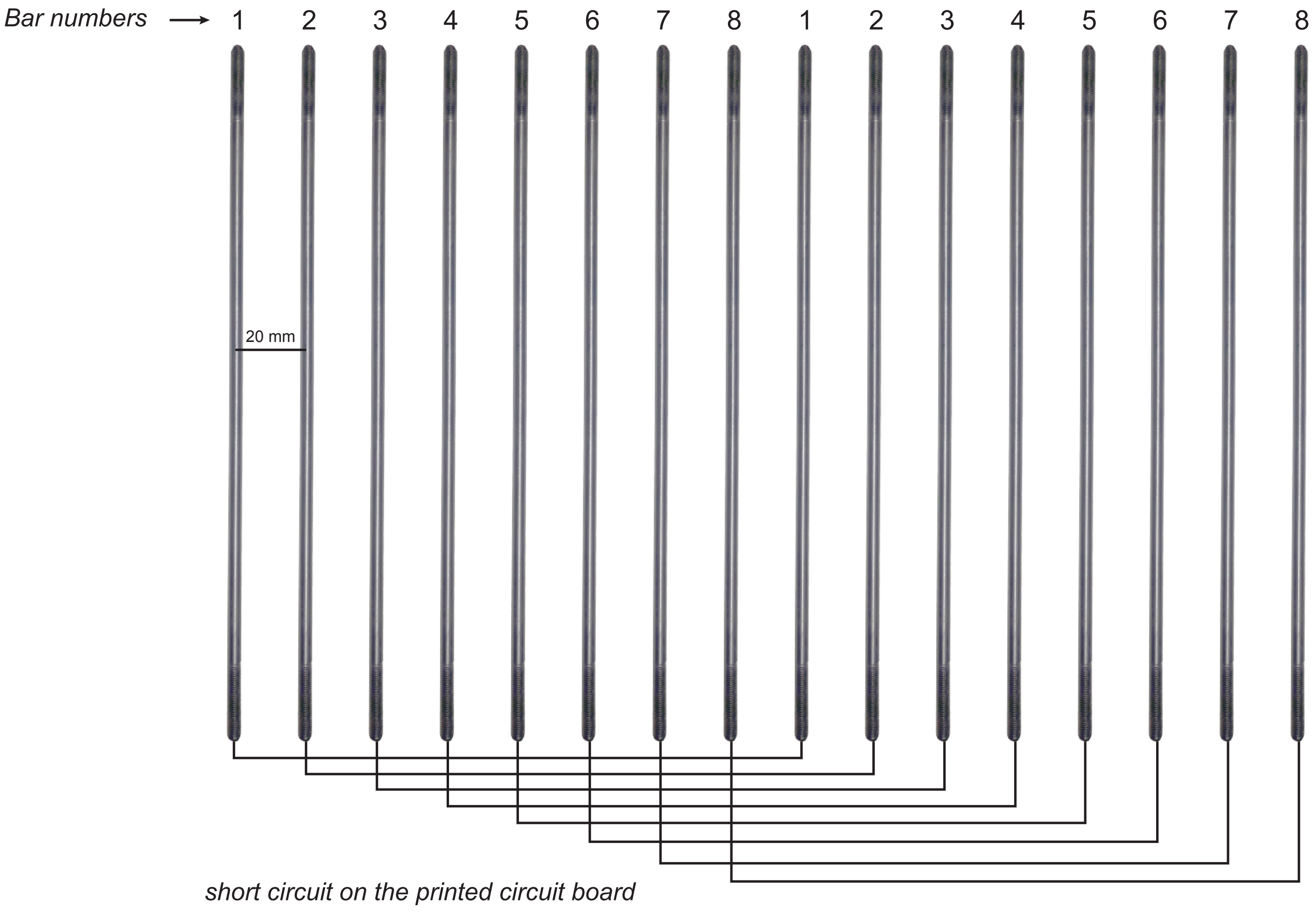

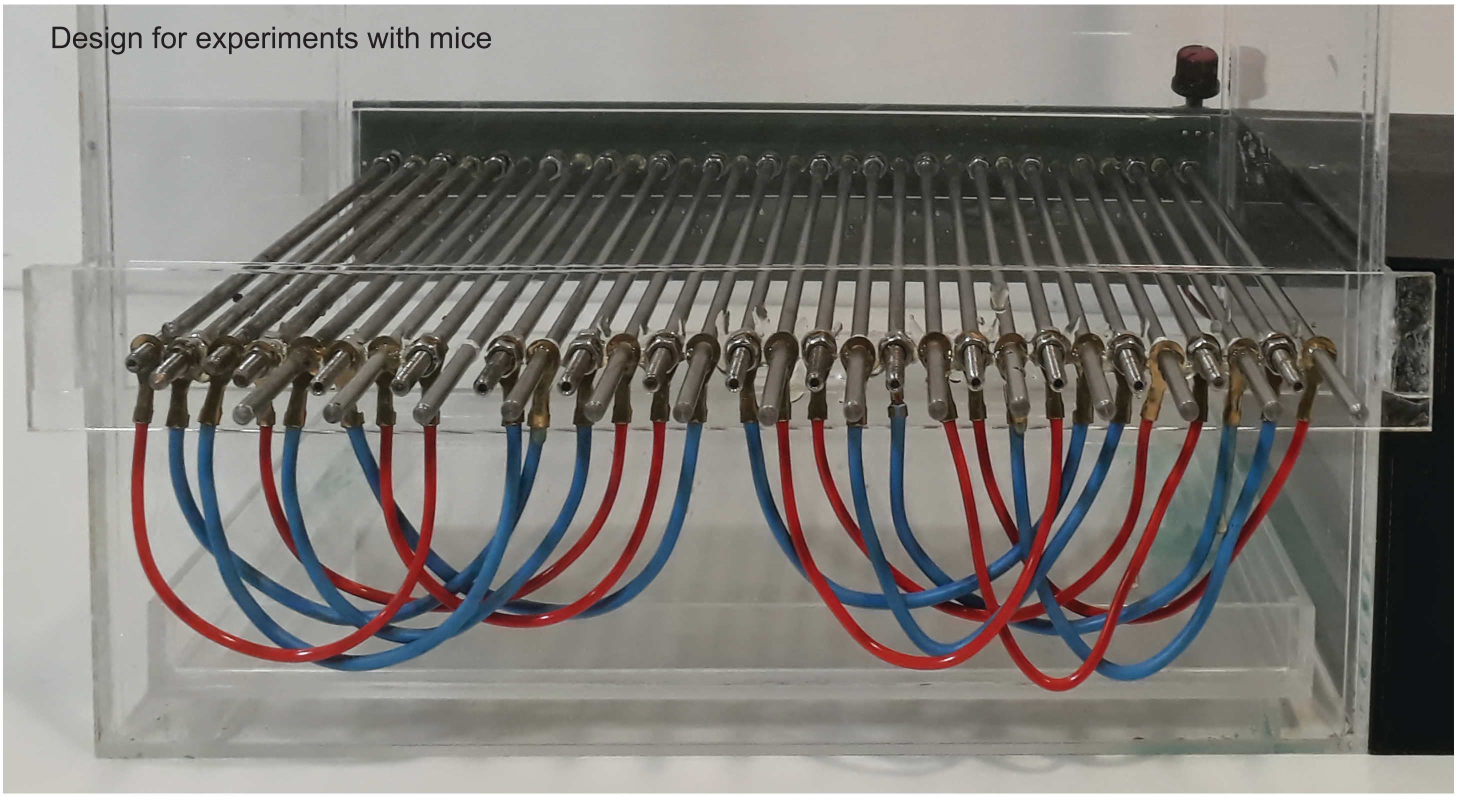

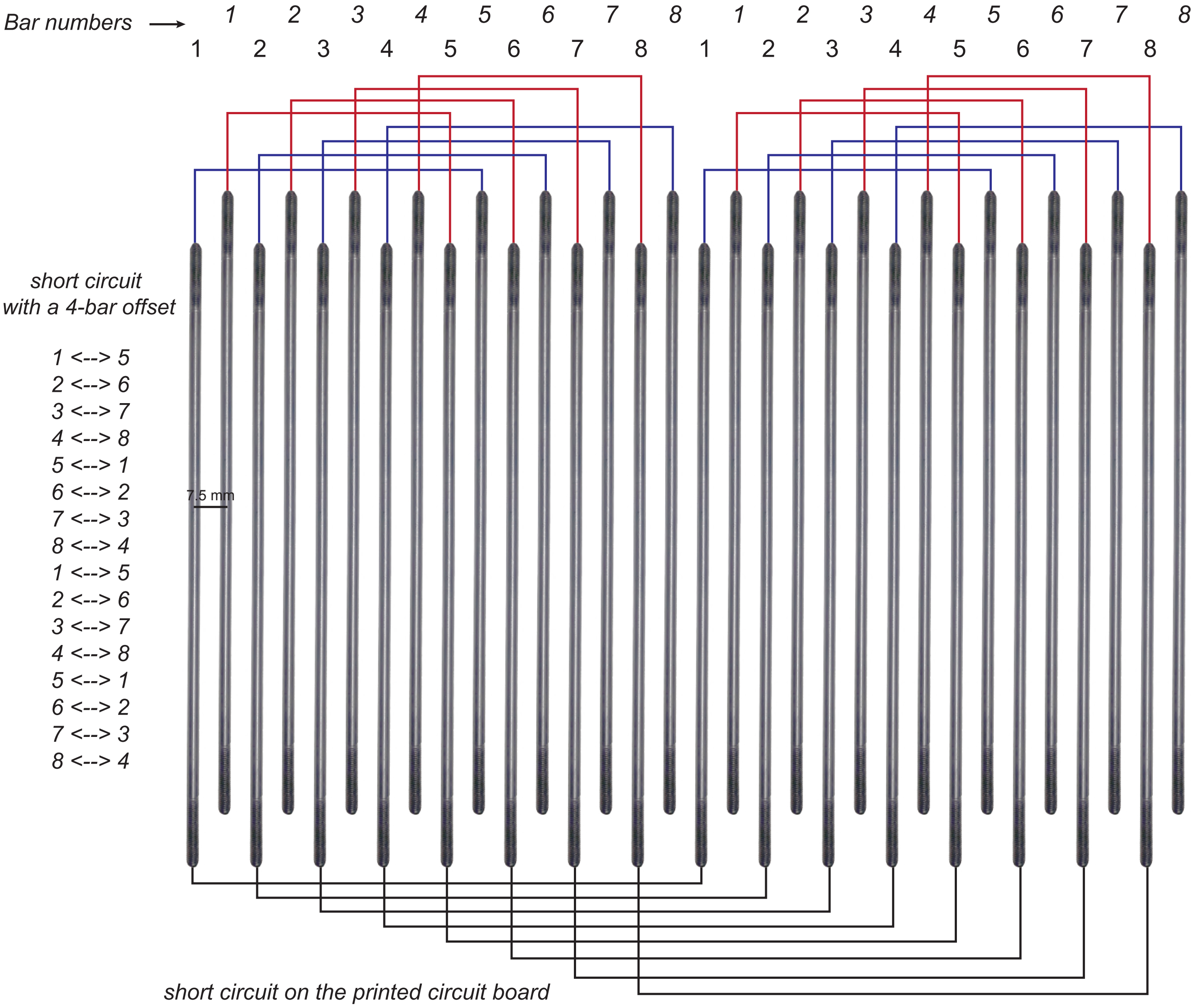

For experiments with mice the number of bars can be increased up to 32 and spaced 7.5 mm from each other. We suggest an external short circuit wired with a 4-bar offset. So that there are no repetitions in an 8-bar sequence covering the full animal extent.

Power Supply

Component	Specification	Quantity
Isolated Transformer	0.03KVA; 127V_{AC}:220V_{AC}	1
Power Supply	1A; 12V	1

Two-layer printed circuit board with control circuit. It has ESP8266-12E footprint and an Arduino Due shield

Component		Quantity
Arduino DUE	https://store.arduino.cc/usa/due	1
Module WI-FI ESP8266-12E	https://www.adafruit.com/product/2491	1
FTDI Serial TTL-232 USB	https://www.adafruit.com/product/70	1

*TOP

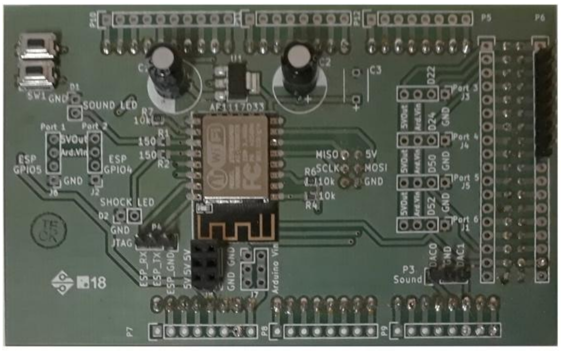

*BOTTOM

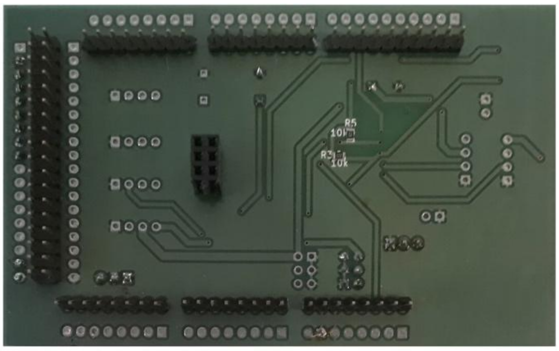

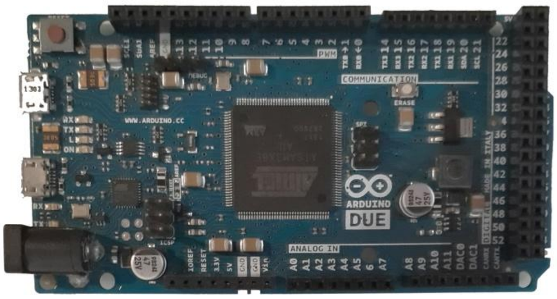

Component	Model	Specification	Quantity
LDO Voltage Regulators	AP1117D33		1
Radial Electrolytic Capacitor		147uF, 550V	2
Resistor SMD		10kΩ 1%	4
Ceramic Capacitor		47uF, 50V	1
SMD Tactile Push button / Key Switch (2 x 6 x 2.5 mm)	KFC-A06		2
Male Pin Headers 2.54mm single (1 x 3 pins)			8
Male Pin Headers 2.54mm single (1 x 4 pins)			6
Male Pin Headers 2.54mm single (1 x 8 pins)			5
Male Pin Headers 2.54mm single (1 x 10 pins)			1
Female Pin Headers 2.54mm single (2 x 3 pins)			1
Dual Pin Headers 2.54mm single (2 x 18 pins)			1

Two-layer printed circuit board with power circuit. Attached to the chamber via 16 mounting holes, this board allows creating electric potential between the conductive bars.

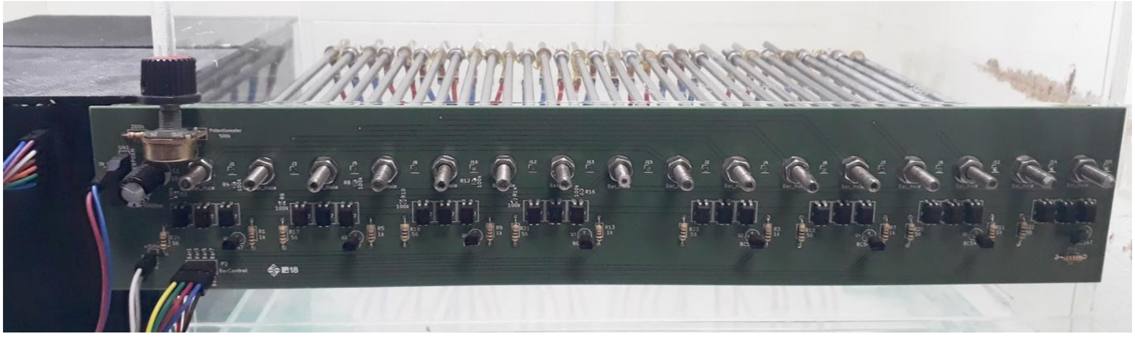

Component	Model	Specification	Quantity
Resistor Through-hole		56Ω 5% 1/4W	8
Resistor Through-hole		1kΩ 5% 1/4W	8
Resistor Through-hole		200kΩ 5% 1/4W	1
Resistor SMD		100kΩ 1% 1/4W	8
Potentiometer -Type B Linear response curve		500kΩ	1
Radial Electrolytic Capacitor		10uF, 350V	1
Bridge Rectifier Diode	2KBP06M	2600V, 2A.	1
Optocouplers	PC817 4-pin	-	24
Transistors	BC547B	-	8
Pin Headers 2.54mm single (1 x 12 pins)			1
Dual Pin Headers 2.54mm single (2 x 4 pins)			1
Pin Headers 2.54mm single (1 x 2 pins)			2
Female Jumper wires			12

Stainless steel cylindrical bar and hex nuts

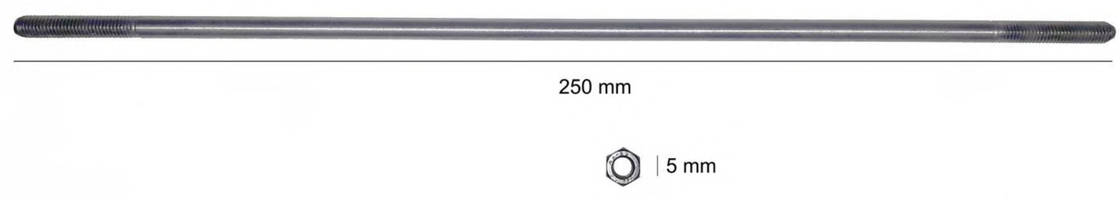

Component		Quantity
Stainless steel cylindrical bars	250 x 5 mm	32 (or 64)
Stainless steel hex nuts	5 mm	64 (or 128)

Printed circuit boards with its components map

Bars circuit

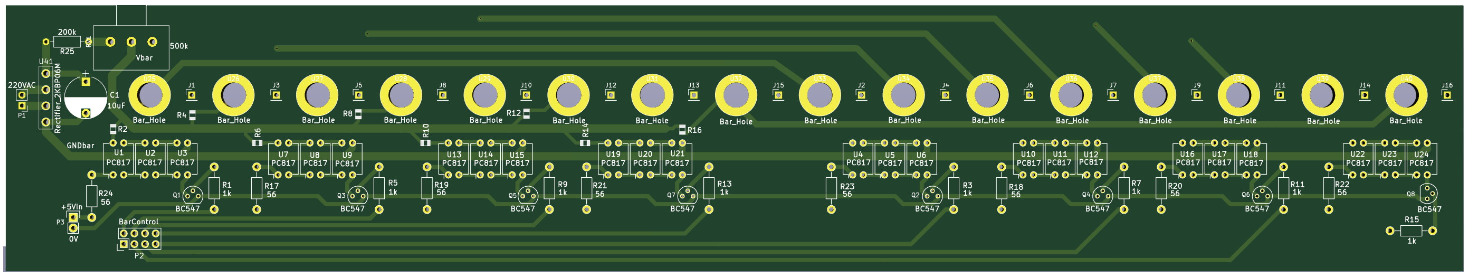

Legend:

. P1 - Isolated transformer input
. P2 - Bar control input
. P3 - Input from Arduino output 5V
. U1 -> U24 - Optocouplers PC817
. U41 - Bridge Rectifier Diode
. Q1 -> Q8 - Transistors BC547B
. R2,R4,R6,R8,R10,R12,R14,R16 - Resistor SMD 100kΩ
. R1,R3,R5,R7,R9,R11,R13,R15 - Resistor SMD 1kΩ
. R17 - R24 - Resistor SMD 56Ω
. R25 - 200kΩ
. C1 - Radial Electrolytic Capacitor 10uF, 350V
. J1 -> J16 - Pins for current test
. *No stamp - upper left corner* - Potentiometer, Type B Linear response curve, 500kΩ

Control circuit to be attached on Arduino shield

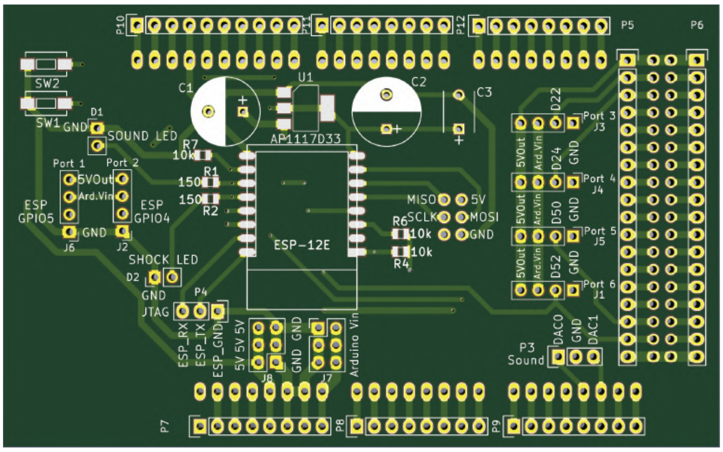

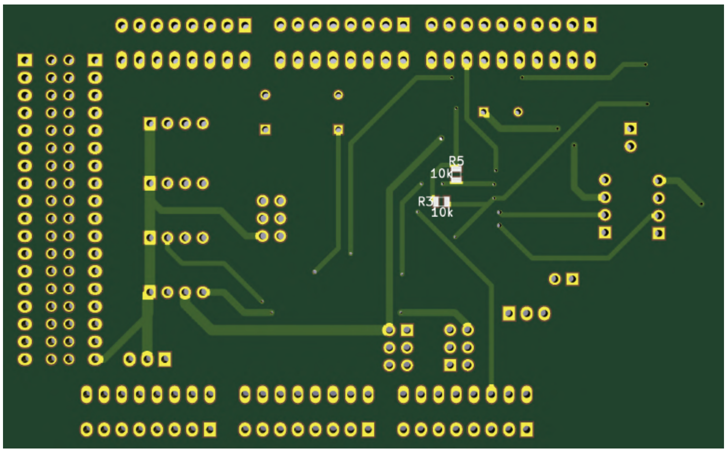

Legend:

. P3 - Arduino Digital-Analogic converter ports for sound output (Default DAC1)
. P4 - Serial port to program ESP8266. GND, TX (Transmit) and RX(Receive)
. P5 -> P12 - Pin Headers that will connect to the Arduino board. Need to be welded underneath.
. Port1 - Port6 - Generic Arduino/ESP8266 outputs (5v out, GND, Arduino in, ESP GPIO04/05, Digital out)
. J8 - Generic Arduino outputs (5v out, GND)
. J7 - Generic Arduino outputs (GND, Arduino Vin)
. D1 - LED signaling sound on
. D2 - LED signaling shock on
. SW1 -> SW2 - SMD Tactile Push button to put ESP8266 into programming mode *(Hold down the GPIO 0 button -- > Press the Reset button -- > Then let go of both buttons).*
*SW1 - GPIO 0
*SW2 - Reset
. R1, R2 - Resistor SMD ~150Ω
. R3 (bottom), R4 (top), R5 (bottom), R6 (top), R7 (top) - Resistor SMD 10kΩ
. U1 - LDO Voltage Regulator
. C1, C2 - Radial Electrolytic Capacitor 147uF, 550V.
. C3 - Ceramic Capacitor 47uF, 50V
. In the center of the circuit to the right side of the ESP8266 - Female Pin Headers that will connect to the Arduino board (MISO/SCLK/5V/MOSI/GND).
Need to be welded underneath.

Wiring diagram

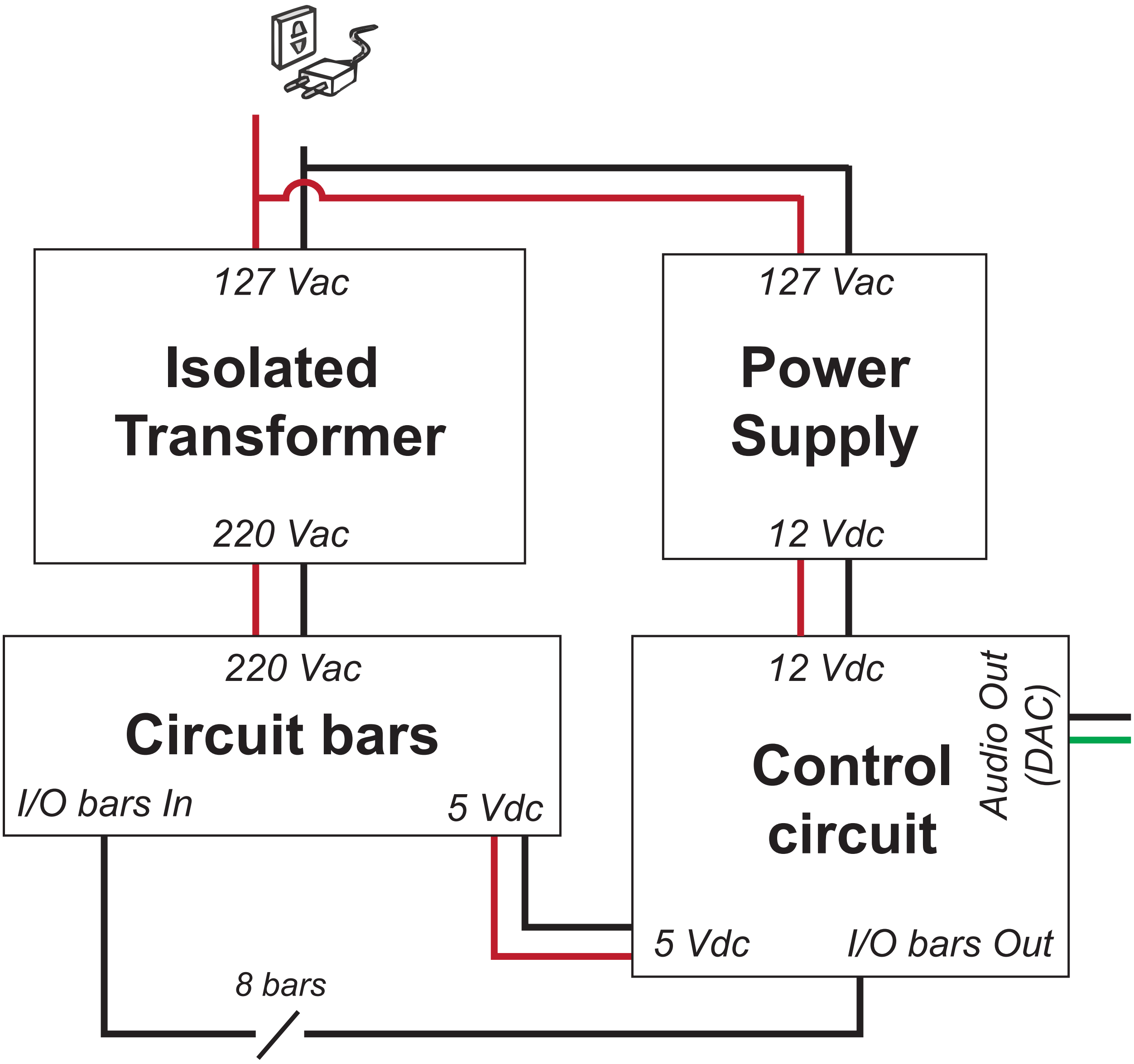

Control Box

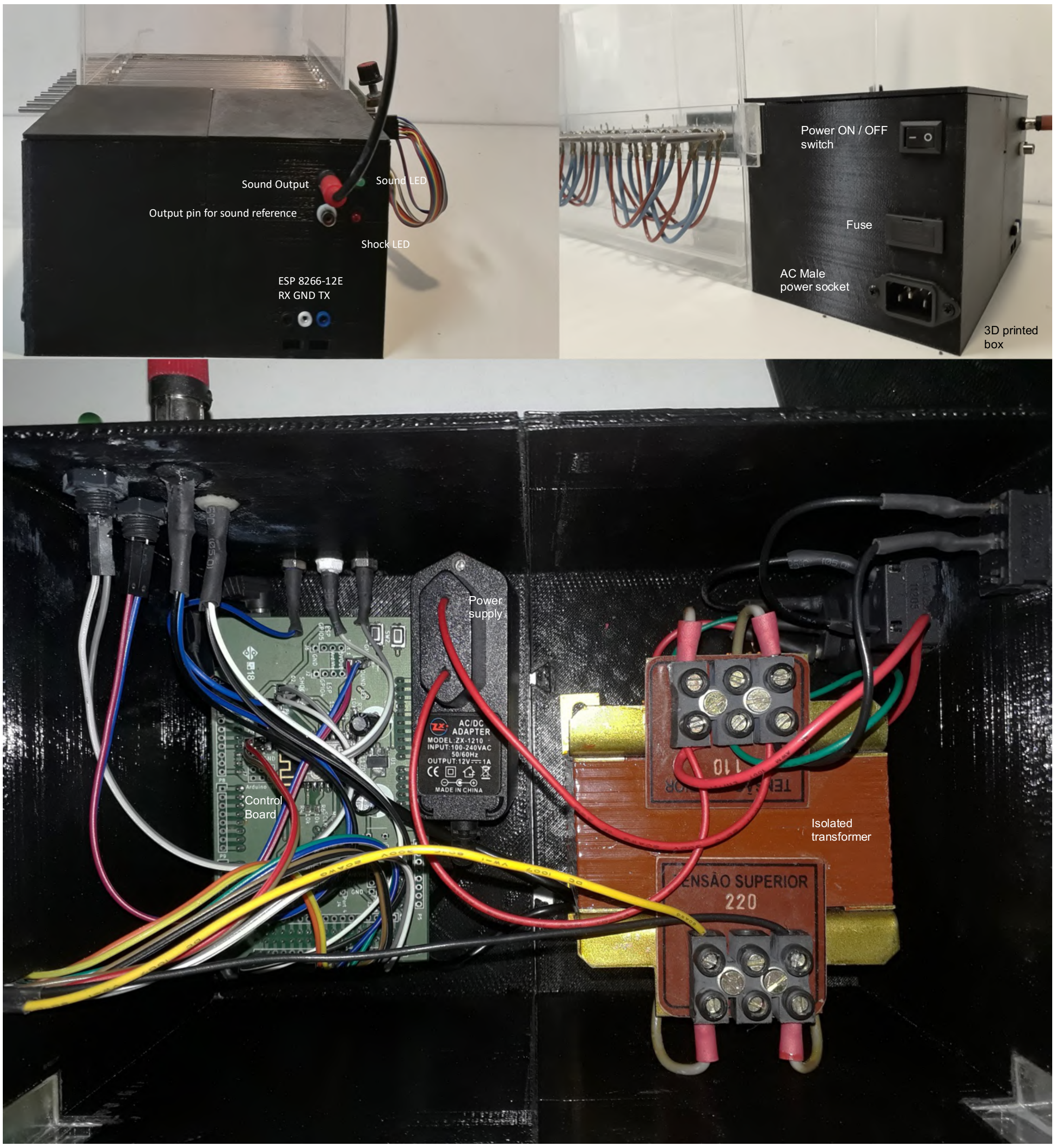

Component		Quantity
Power ON/OFF switch	18.4 x 11.6 mm	1
AC Male computer power socket	Standard	1
Fuse Socket	-	1
Fuse 1A	-	1
Female RCA connector	-	2
Led	5 mm	2
Female banana connector	4 mm	3
Dual Pin Headers 2.54 mm single (2 x 6 pins)		1
Female Jumper wires		12
